# The influence of stormwater infiltration on downslope groundwater chemistry

**DOI:** 10.1007/s10653-023-01732-3

**Published:** 2023-08-30

**Authors:** Meenakshi Arora, Timothy D. Fletcher, Matthew J. Burns, Andrew W. Western, Chui Fern Yong, Peter J. Poelsma, Robert B. James

**Affiliations:** 1 Faculty of Engineering and Information Technology, University of Melbourne, Parkville, Victoria 3010 Australia; 2 School of Ecosystem and Forest Sciences, University of Melbourne, 500 Yarra Boulevard, Burnley, VIC 3121 Australia

**Keywords:** Infiltration basins, Stormwater, Vegetation, Legacy contamination, Subsurface infrastructure

## Abstract

Stormwater infiltration basins have been used extensively around the world to restore urban hydrology towards more natural flow and water quality regimes. There is, however, significant uncertainty in the fate of infiltrated water and accompanying contaminants that depends on multiple factors including media characteristics, interactions with downslope vegetation, legacy contaminants, and presence of underground infrastructure. Understanding the influence of such factors is thus central to the design and siting of infiltration basins. An extensive field program was established to collect monthly data on ground water quality, including nutrients and major ion concentrations, in a bore network downstream of a stormwater infiltration basin in Victoria, Australia. The groundwater samples were analysed for temperature, pH, EC, turbidity, major ions (Na^+^, Ca^2+^, K^+^, Mg^2+^, Cl^−^, SO_4_^2−^, NO_3_^−^, CO_3_^2−^, HCO_3_^−^), NOx and heavy metals. The collected data were used to understand the origin and fate of water and solutes in the subsurface and their interactions with the soil matrix. The results revealed that Ca–HCO_3_, Na–Cl water types predominate in the study area, grouped in 3 clusters; shallow fresh groundwater in the vicinity of the basin (near basin), deep saline groundwater further downstream of the basin (near-stream) and a mid-section where rock-water interaction (Na–HCO_3_ water) through cation exchange control the chemistry of groundwater. The results also suggest that as the water moves downstream of the basin, it experiences significant evapotranspiration and concentration due to the presence of deep-rooted vegetation. The results suggest that while infiltration basins can remove infiltrated contaminants, the infiltrated stormwater can mobilise legacy contaminants such as nitrate. Overall, the efficacy of infiltration basins in urban regions depends substantially on the downstream vegetation, urban underground infrastructure and the presence of legacy contaminants in the soils. These all need to be considered in the design of stormwater infiltration basins.

## Introduction

Urban stormwater is recognised worldwide as a primary cause of the degradation of receiving waters, depriving urban communities of the ecosystem services that are provided by healthy urban streams. The degradation occurs as a result of changes to flow regimes and associated water pollution (Walsh et al., 2005). Urban streams typically have much more ‘flashy’ flows, with frequent large runoff peaks, which transport contaminants, cause erosion, and degrade stream channels and their habitat value for in-stream biota (Booth & Jackson, 1997). Urban drainage not only increases peak flows during and following storms; the creation of impervious areas and hydraulically efficient drainage networks deplete groundwater stores and subsequent stream baseflows (dry-weather flows), depriving aquatic organisms of physical habitat (Bhaskar et al., 2016; Price, 2011). Baseflows are critical to stream health; loss of baseflow causes habitat loss and local extinction of aquatic fauna (Poff et al., 1997). There are substantial efforts internationally (Walsh et al., 2016) to mitigate these impacts, using a range of technologies such as stormwater wetlands, rain-gardens and infiltration basins (Fletcher et al, 2015).

Infiltration basins are among the most widely applied stormwater control measures worldwide, in part for their ability to intercept stormwater runoff and allow it to infiltrate into the ground, with the assumption that this will recharge groundwater and also help restoring clean, filtered baseflows to urban streams (Hamel et al., 2013). Stormwater infiltration basins provide substantial localised (point-source) additions to the subsurface water store, with the potential to generate major contributions to groundwater and lateral seepage to streams. There is increasing concern (Bhaskar et al., 2016), however, that infiltration basins may have unintended consequences both in terms of flow regime and water quality, due to the gross disturbance of subsurface flow paths caused by urban underground infrastructure (e.g. water, sewer & gas pipes, communications conduits) and their associated gravel-filled trenches for underground pipes, cabled ducts, etc. (collectively referred to as the ‘urban karst’ (Kaushal & Belt, 2012)). These highly-permeable trenches potentially lead to ‘short-circuits’ that could rapidly transmit water and associated contaminants (Roy & Bickerton, 2012) to streams, undermining the objectives of infiltration systems.

The potential for transfer of contamination from infiltration basins to surrounding groundwater has been identified, although such risks vary with the characteristics of the contaminants themselves, the nature of the infiltration substrate, and the characteristics of the vadose zone and aquifer surrounding the infiltration system (Pitt et al., 1999). The risks tend to be highest for highly mobile contaminants such as salts, nitrates and even some heavy metals such as zinc and nickel (Aryal et al., 2007; Behroozi et al., 2021; Clark & Pitt, 2007). Stormwater infiltration has also been shown by Foulquier et al. (2009) to have the potential, in some situations, to result in thermal pollution of nearby aquifers.

Infiltrated water could also interact with legacy contaminants. The legacy contamination might include leakage from sewers, old landfill sites, discharges from past industrial processes, or other disturbances to natural soil due to construction activities (Roy & Bickerton, 2010, 2012). The enhanced movement of subsurface water, resulting from infiltration of stormwater, might mobilise some of these resident contaminants in the subsurface. In doing so, stormwater infiltration systems might not achieve their aim of providing cleaner baseflow, but instead lead to groundwater contamination and more polluted baseflows. Additionally, over time the filter media reaches its maximum sorption capacity and if not properly maintained (replaced or regenerated), the basin itself can become source of contamination (Behbahani et al., 2020).

Given this potential for contamination, there is a critical need to understand the fate of both infiltrated and legacy contaminants in the urban soil to ensure that we achieve the best outcomes from the stormwater infiltration basins. To bridge this gap, in this paper we aim to assess the influence of stormwater infiltration on the chemistry of downslope water quality. We do this using solute composition analysis in the infiltration basin, as well as in a bore network downstream of the basin, leading to a downslope stream which receives baseflows from these groundwaters. The work provides insights into the types of groundwater chemistry changes that may result from long-term stormwater infiltration and can be used to provide insight into likely ecological consequences for receiving waters, be they groundwater or streams.

## Materials and methods

### Experimental design and site description

This study was conducted on a large-scale stormwater infiltration basin (hereafter ‘the basin’) which is located at the foothills of the Dandenong Ranges, in the outer eastern suburbs of Melbourne, Australia. Temperate climatic conditions occur at the study site (mean annual rainfall and potential evapotranspiration of 730 mm and 1030 mm, respectively (Bonneau et al., 2018)). The monthly distribution of rainfall is reasonably uniform—albeit with a slight winter-spring bias—and there is an excess of precipitation over evapotranspiration in the cooler months, meaning groundwater recharge is limited to this period. The underlying soils at the site are sand clay loam composed of quartz–kaolinite–muscovite, 74% quartz and 26% of all clay types combined (Behroozi et al, 2021) with saturated hydraulic conductivity 0.1–1.1 mmhr^-1^, at least 4–5 m deep, and sit on weathered rhyodacite.

**Fig. 1:**
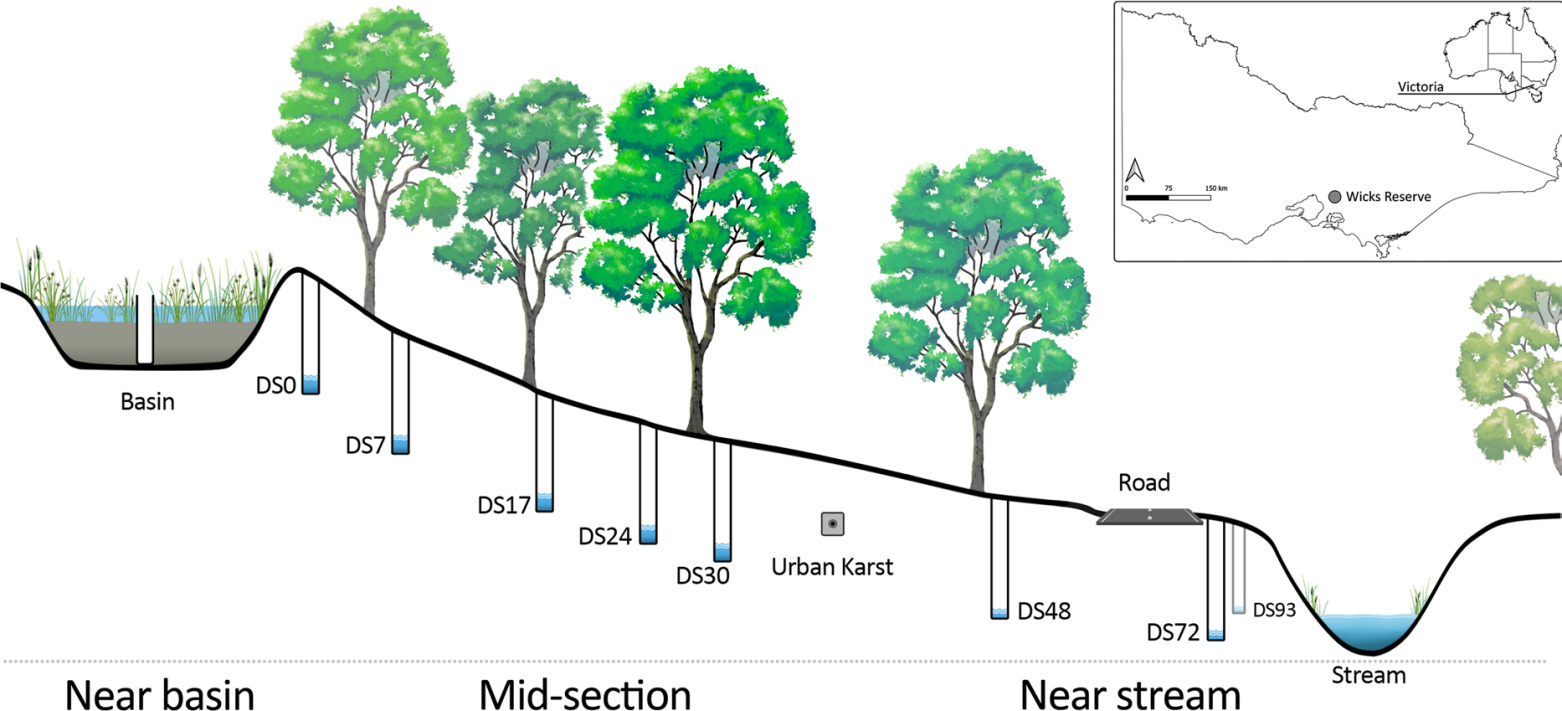
Conceptual diagram and map showing bore location in relation to the infiltration basin and stream

The basin was constructed in 2011 and drains a catchment made up of ~ 5 ha of impervious surfaces—house roofs, driveways, and roads. The basin has a surface area of ~ 1800 m^2^ and comprises a 0.8 m deep filter (0.4 m loamy sand-based soil overlaying 0.4 m of scoria gravel, with two transition layers of sand to bridge the difference in particle size between the loamy sand and scoria layers). It is also densely planted with a mix of swamp grasses and rushes (*Centella cordifolla*, *Amphibromus nervosus, Eleocharis acuta*), sedges (*Carex appressa*) and shrubs (*Acacia vertillata*, *Epacris impressa*). Stormwater stored in the basin exfiltrates into the surrounding soils at a rate of approx. 1000 m^3^ month^−1^ and flows down the hillslope towards the local receiving water (Dobsons Creek, 100 m away). The hillslope itself is dominated by mature, deep-rooted Eucalyptus species (primarily *Eucalyptus ovata* and *Eucalyptus obliqua*), with a dense understory vegetation comprised of ferns and shrubs (Fig. 1).

There is historical evidence that the site has been artificially drained in the past to make it suitable for sporting activities (Bonneau et al., 2018). This is also supported by the finding of asbestos slotted drainage pipes during basin construction. Some of the site could also have been used for agriculture in the early twentieth century. In addition, there is a sewer pipe, surrounded by a gravel trench, running perpendicular to the basin between it and the receiving water. Combined, these lines of evidence suggest that there is some potential for legacy pollutant impacts at the site.

The spatial and temporal water quality patterns_2width="709" imgheight="32 were used to identify the source of water, fate of infiltrated water and solute and its interaction with the aquifer material through a combination of graphical and correlational techniques.

### Sample collection and analysis

Monthly water samples were collected from the basin and bore network downstream of the infiltration basin from July-2018 to May-2019. Each sampling event was numbered with July 2018 as event 6, August 2018 as event 7 and so on. The bore network extended approximately 100 m downstream of the basin, across the sewer pipe trench and to the receiving water (Fig. 1). We represent the site using two letters and two numbers—DS represents downstream of basin and the numbers represent distance from the basin in metres, e.g. DS07 is a bore 7 m downstream of the basin. At some locations, there are 2 bores at same distance, such as 17 East and West represented as DS17 E and DS17 W. Two bores within the sewer trench (urban karst) are labelled ST East and ST West. Prior to sampling, each bore was pumped continuously to purge at least three casing-volumes of water from the bore. The sample water was filtered through a 0.45 µm syringe filter and collected in 15 mL conical polypropylene centrifuge tubes that were rinsed three times with the filtered sample water. Field parameters were measured at each site using a YSI 6920 Multiparameter Water Quality Probe (Sonde) linked to a YSI 650 display unit. The probe measured water temperature, pH, EC, dissolved oxygen and turbidity, and was calibrated prior to each sampling trip to ensure accurate measurements.

Collected bore water samples were transported to the laboratory in an insulated container and stored at 4 °C until analysis. All the samples were then analysed for chemical parameters including major cations (Na^+^, Ca^2+^, K^+^, and Mg^2+^), major anions (Cl^−^, SO_4_^2−^, NO_3_^−^, CO_3_^2−^ and HCO_3_^−^), nutrients and some heavy metals (ferum, zinc, barium, manganese, aluminium, copper, nickel and strontium). The chemical ground water quality parameters were analysed using the standard methods for the examination of water and wastewater and results are presented in Table 1. The major cations (Na^+^, Ca^2+^, K^+^, and Mg^2+^) were analysed using an Inductively Coupled Plasma-Optical Emission Spectrometer (ICP-OES) (Optima 4400, Perkin Elmer, USA) and major anions (Cl^−^, F^−^, SO_4_^2−^, NO_3_^−^) were analysed using Dionex IC-5000 Ion Chromatograph. The detectable limit of the ICP/IC was set at < 0.001 mg/l for the cations/anions. NIST (National Institute of Standards and Technology) certified reference standards were used for calibration of ICP and IC. The concentrations of carbonates and bicarbonate were determined by the acid titration method for alkalinity as well as by using the ion balance equation and the concentrations calculated by two methods were within 5% error range. Nitrate levels were analysed as per the standard methods for the examination of water and wastewater (WPCF, 2005) and the heavy metals were analysed using standard method WG020A at an external laboratory, Australian Laboratory Services (ALS). All the chemicals used for preservation and analysis were of analytical reagent grade.

**Table 1:** Water quality recorded at various sampling locations in October 2018

		Concentration in mg/l									
Sample ID	EC	Iron	Zinc	Barium	Manganese	Aluminium	Copper	Nickel	Strontium	Fluoride	Nitrate
Basin	0.343	0.41	0.052	0.014	0.140	0.130	0.009	0.014	0.024	0.15	0.057
DS0	0.161	0.07	0.044	0.053	0.011	0.030	0.015	0.002	0.003	0.025	0.04
DS7	0.149	0.05	0.013	0.035	0.002	0.030	0.002	–	–	0.025	0.13
DS17	0.305	0.85	0.043	0.074	0.023	0.050	0.009	0.008	0.003		
DS 17East	–	0.06	0.076	0.150	0.029	0.010	0.006	0.002	0.005		
DS 17West	0.169	0.79	0.011	0.047	0.005	0.020	0.002	–	–	0.025	0.002
DS 24	0.840	1.60	0.006	0.150	0.062	–	–	0.001	0.058		
DS 30	0.669	0.02	0.031	0.089	0.410	–	0.004	0.001	0.029		
ST East	–	0.24	0.043	0.072	1.100	0.080	0.008		0.031		
ST West	0.74	2.50	0.016	0.130	0.130	–	–	0.001	0.063	0.025	4.7
DS 48	–	0.97	0.440	0.260	0.280	–	0.018	0.002	0.130		
DS 72	3.956	–	0.650	0.310	0.069	–	0.017	0.004	0.240	0.28	7.8
DS 93	1.482	0.11	0.080	0.170	0.016	0.090	0.003	0.006	0.110	0.14	0.009
Dobsons Creek	0.268	0.26	0.003	0.053	0.013	0.020	0.001	–	0.051	0.09	0.46
Potable water	0.06	0.04	0.028	0.015	0.001	0.030	0.054	–	0.018	0.81	0.17
Sewer			0.052							0.74	0.08

### Data analysis

As groundwater quality depends on multiple factors including climate, soil type, aquifer material, vegetation cover and the quality of infiltrated stormwater, it is important to understand the source of water in the subsurface as well as the processes that control the fate and movement of infiltrated water and solutes (contaminants) for optimal management of water quality. In this study therefore, we used the spatial and temporal water quality patterns and a combination of graphical and correlational methods (Table 2) such as Piper diagrams (source of water), Stiff pattern diagrams (variation in chemical composition over space and time), Gibb’s diagrams (dominant mechanisms), and various ionic ratios to understand the interaction between infiltrated water, the aquifer material and other environmental factors.

**Table 2:** Various data analytical methods used

Analysis type	Information provided
Piper diagrams	Source of water
Stiff pattern diagrams	Variation in chemical composition over space and time
Gibb’s diagrams	Dominant mechanisms
Ionic correlations	interaction between infiltrated water, the aquifer material and other environmental factors

#### Water type (source) and hydrochemical facies: Piper diagram

We plotted a Hill and Piper diagram for each bore to provide insights into the relationship between different dissolved solutes and water type/source. These plots consist of three components and present anion and cation data in two separate triangles and the combined cation and anion field in a diamond plot to provide an overall assessment of the hydrochemical characteristics of a water sample. The Piper plots provide insights into spatial variability in groundwater quality.

#### Key Processes/mechanisms: Gibb’s diagram

Gibbs diagrams were plotted to understand the relationship between collected groundwater chemistry and aquifer lithological characteristics (Gibbs, 1970). The plots show three distinct fields; precipitation dominance, evaporation dominance and rock–water interaction dominance to identify the major processes controlling groundwater chemistry at each of the sampled sites over the sampling period.

#### Chemical composition: Stiff pattern diagram

The Stiff diagrams provide greater insights into temporal variability in ionic composition of water samples collected over the sampling period. Therefore, Stiff diagrams were plotted for each bore to identify the differences and similarities in ionic composition over over time and space including; (i) change in ionic composition in each bore over time and (ii) change is ionic composition at each bore between the point of infiltration (basin) to the local receiving water (Dobson’s creek). A vertical line in the middle presents the zero axis from which major ions were plotted on four parallel horizontal axes extending on each side of the vertical line. The four major cations (Na^+^, K^+^, Ca^2+^ and Mg^2+^) were plotted to the left of the vertical axis and four major anions (Cl^−^, SO_4_^−^, HCO_3_^−^ and CO_3_^2−^) on the right side. The points on each side were then connected to form an irregular polygonal shape. Each shape represents a different chemistry of water and the width of the shape indicates the ion concentration. Diagrams with similar shapes indicate similar hydrochemical properties and origin. The change in shape over time indicates the variability in water quality.

#### Ionic correlations

To further understand the processes that might have played an important role in influencing the groundwater chemistry during its travel in the subsurface, we used several commonly used correlations, including:

i) Chloro-alkaline indices-CAI (Schoeller, 1977) to indicate the direct base exchange (exchange of Na^+^ and K^+^ ions from water with Mg^2+^ and Ca^2+^ ions of the rocks) or indirect base exchange (exchange of Mg^2+^ and Ca^2+^ from the water with Na^+ ^and K^+^ ions from rocks) by calculating the CA-I using Eq. 1, where all the concentrations are in meq/l;

1 $${\text{CAI}}{-}{\text{I }} = \, \left[ {{\text{Cl}}^{ - } - \left( {{\text{Na}}^{ + } + {\text{K}}^{ + } } \right)} \right]/{\text{Cl}}^{ - }$$

ii) Na/Cl ratio; and.

iii) Bicarbonate + carbonate/Cl ratio.

## Results and discussion

Temperature of groundwater in the area ranged from 10 to 25 °C throughout the study period and was spatially uniform, so we do not expect any influence of the temperature on the observed spatial trends in water quality. The pH ranged between 5 and 6 for all sites except for the basin, DS24, ST West, DS48, DS72, Dobsons creek and potable water (these sites were closer to neutral, with pH between 6 and 7). This shows that the water in the area is predominantly acidic, which can be attributed to weathered rhyodacite soils. The EC ranged from a low of 0.1–0.9 mS/cm for most of the bores to a high of 1.0 and 4.0 mS/cm for sites closer to the stream (DS 93 and DS 72 respectively), suggesting some form of enhanced evaporation. Very high levels of nitrate were reported in DS72 (7.8–74.13 mg/l) throughout the sampling period. DS 48 (0.001–2.05 mg/l) and DS93 (0.001–0.4 mg/l) also displayed higher nitrate levels compared to all other bores, where nitrate levels were well within the permissible limit of 50 mg/l (Australian Drinking Water Guidelines, 2011). The concentration of all the tested metals was very low in the tested samples and fluoride concentrations in all the samples were within the permissible range (Australian Drinking Water Guidelines, 2011) as presented in Table 1. The major ions results are discussed in detail in the following sections.

### Water type (source) and hydrochemical facies: Piper diagram

**Fig. 2:**
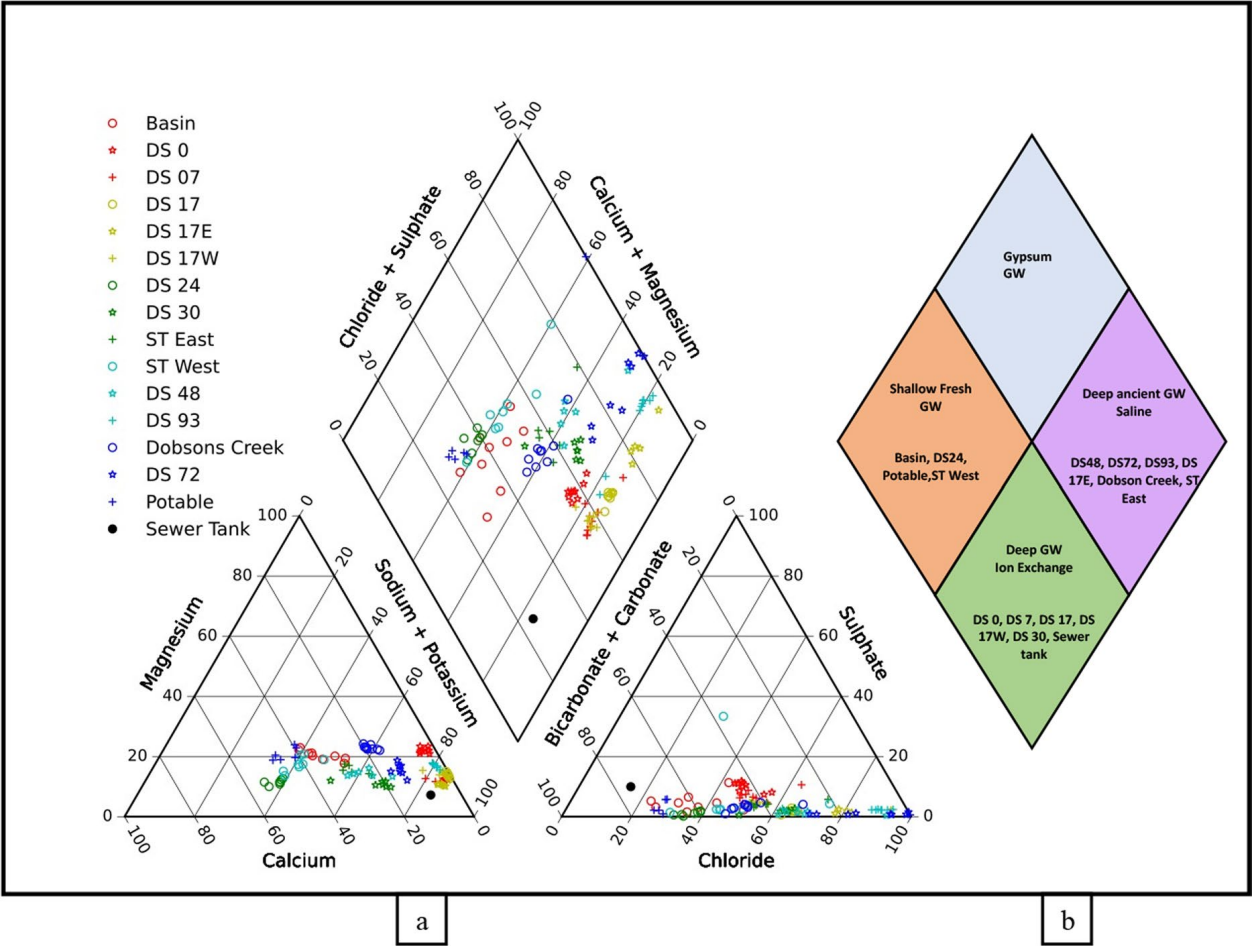
Piper diagram with hydrochemical facies of **a** Basin, all sampled bores and Dobsons creek **b** Water type for each sample

Figure 2a presents the geochemical analysis for the water collected from the basin, all the bores, and the Dobsons Creek for the entire sampling period in the form of piper trilinear diagram. In the figure, the major cations are presented on the lower left triangle while anions are presented on the lower right triangle. The hydrochemical facies based on both anions and cations are plotted in the top diamond. As the diamond plot provides the overall information about the source of water and the hydrochemistry, Fig. 2b presents a combined diamond plot for all the bores and their corresponding hydrochemistry (type of water) based on the position in the diamond.

**Fig. 3:**
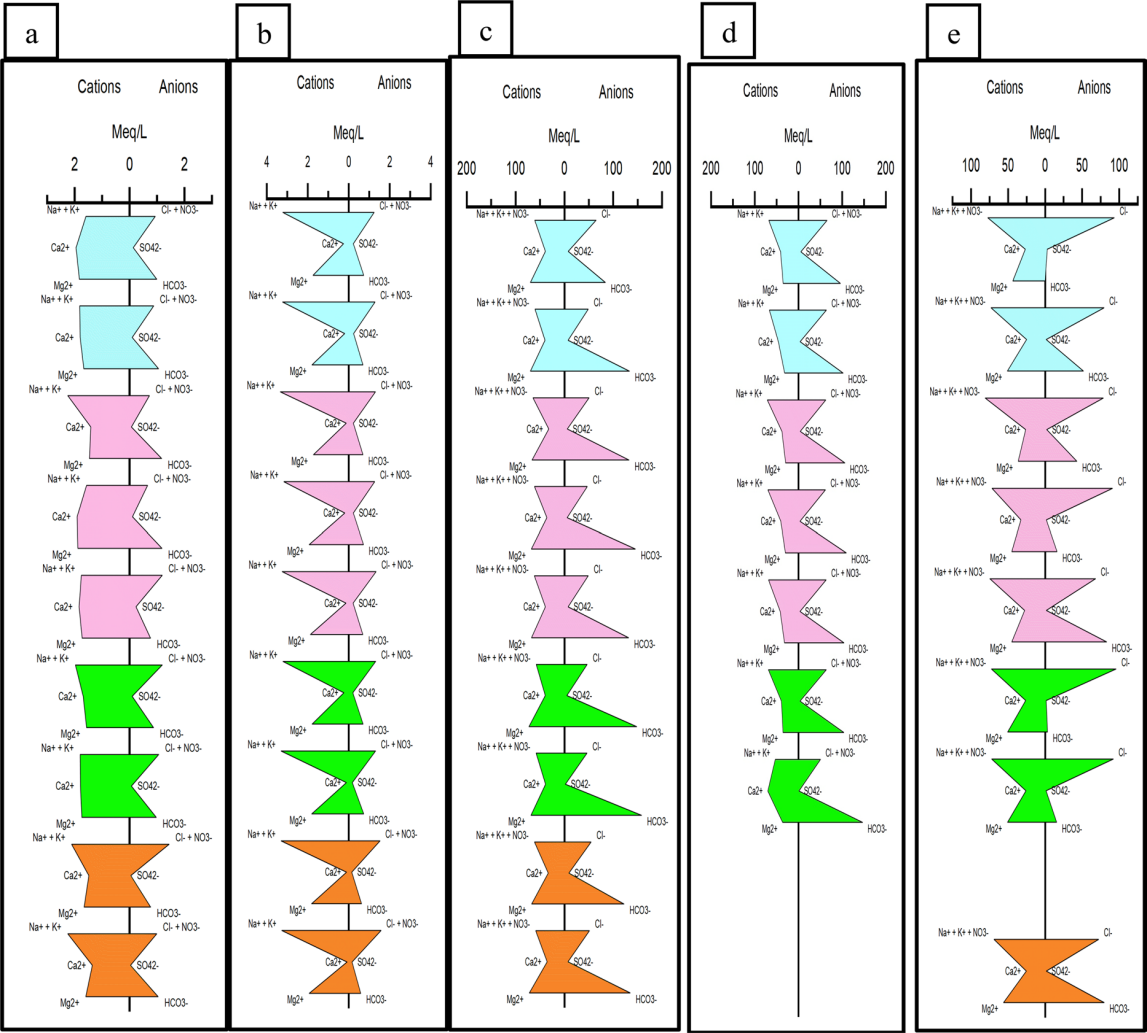
Stiff representing the different water chemistry for selected bore overs different seasons; Blue July–Aug (Winter), Pink Sept–Nov (spring), Green Dec–Feb (Summer) and Orange March–May (Autumn); **a** Basin **b** DS0 **c** Dobsons creek **d** DS30 **e** DS72

The diamond plot for the infiltration basin (Fig. 2) show that all the collected samples are clustered in the calcium bicarbonate type. The results from bore network revealed three main clusters: (i) a near basin cluster with calcium bicarbonate dominated water type (potable water, basin, DS 0, DS24, and ST West), indicating a freshwater source and more reflective of stormwater infiltration in the basin and immediately downstream of the basin; (ii) a near stream cluster with Na–Cl dominated water indicating evapo-concentration due to large trees (DS 48, DS 72, DS 93, ST East and DS 17E) in the region downstream of sewer trench and closer to stream (Western et al., 2021) except bore 17 E.; and (iii) an intermediate cluster on the border line indicating a combination of evapo-concentrated groundwater and the interaction with soil matrix through ion exchange (DS 7, DS 17, DS 17W, DS 30 and the Sewer tank) in the region between the basin and the sewer trench. Water sampled from the Dobsons Creek displayed a similar characteristic to the near stream bore cluster (Na–Cl dominated water). Alkali metal cations (Na + K) in the majority of the groundwater samples exceed Alkaline earth metals (Ca + Mg) indicating Ca and Mg from the infiltrated water are being exchanged with Na/K from the clay (Fig. 3).

### Key processes and mechanisms: Gibb’s diagram

**Fig. 4:**
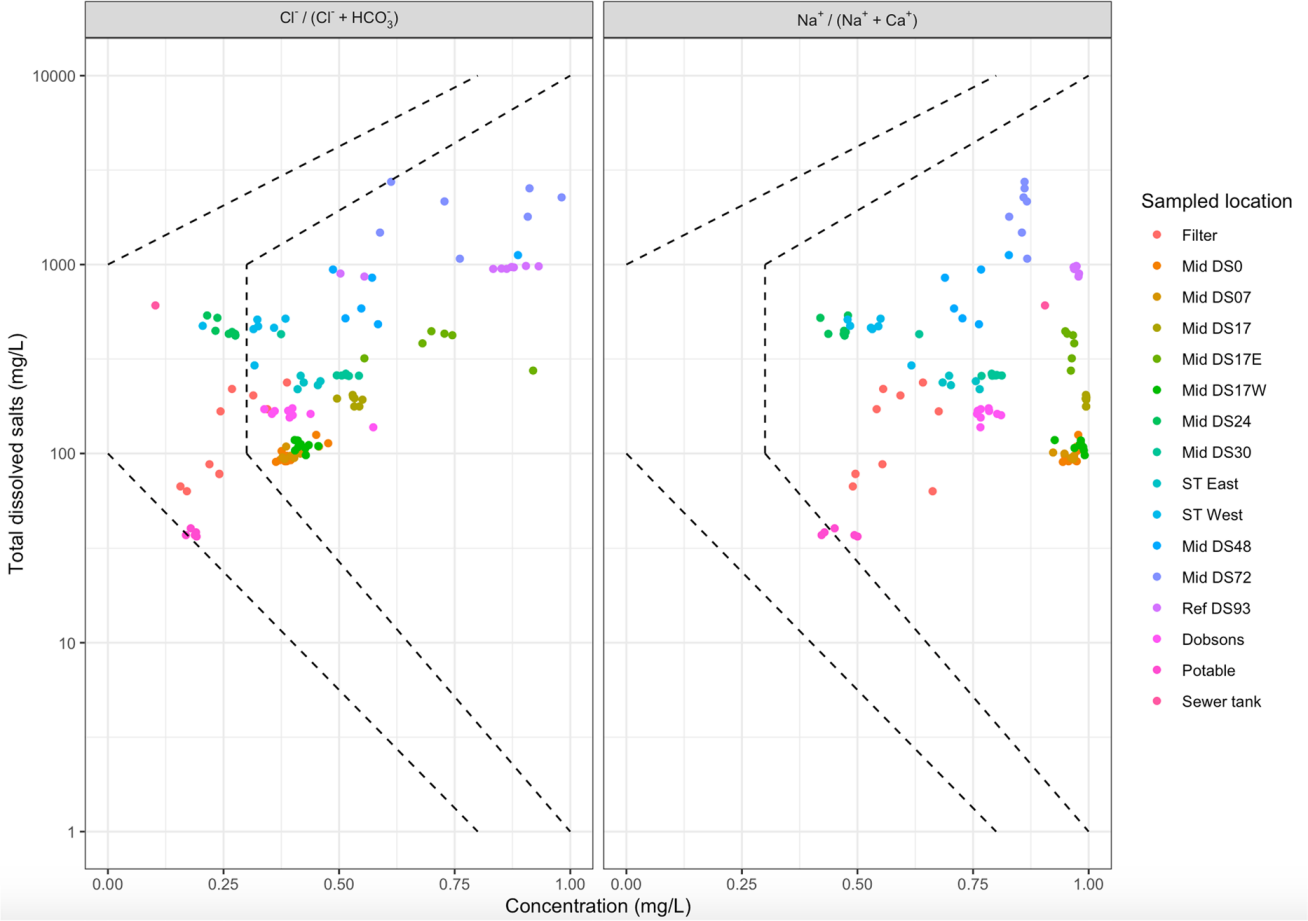
Gibbs diagram **a** (Clˉ/(Clˉ + HCO3ˉ) versus TDS and **b** (Na^+^ + K^+^/(Na^+^ + K^+^ + Ca^2+^) versus TDS

**Fig. 5:**
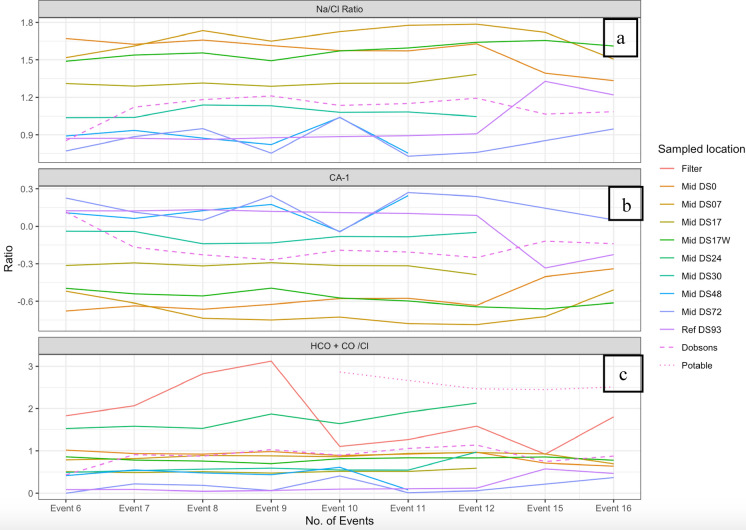
**a** Na/Cl ratio **b** CA-1 plots and **c** HCO_3_ + CO_3_/Cl plots for all the sampled bores

**Fig. 6:**
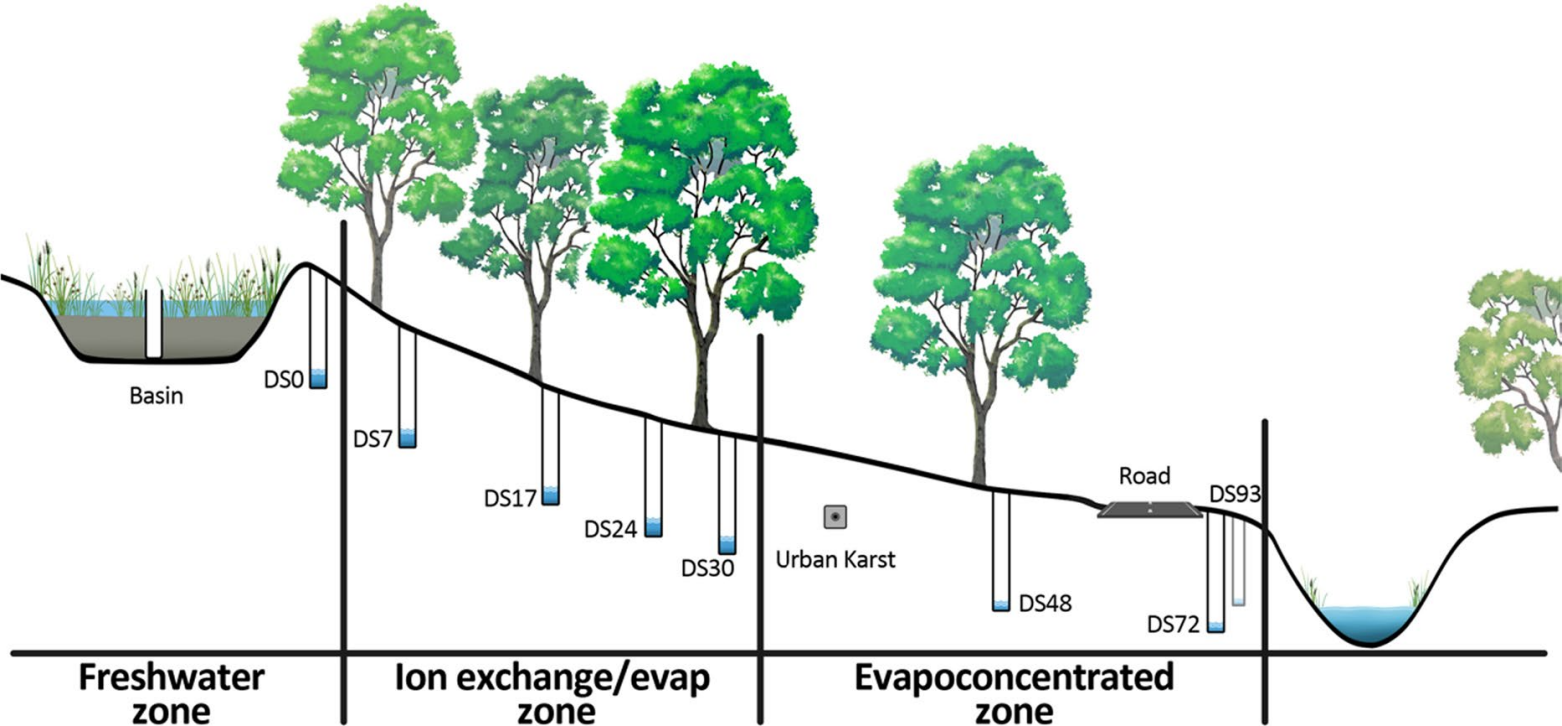
Schematic diagram showing clusters based on water chemistry

The Gibbs diagram was used to determine the dominant mechanisms controlling groundwater hydrochemistry across the site for the study period (Fig. 4). The plots revealed that chemical weathering of subsurface rocks was the main controlling factor governing the groundwater quality in the area, along with some contribution from evaporation and freshwater infiltration (rainfall). Figure 4a and b reveal three distinct clusters wherein potable water and infiltration basin water quality can both be attributed to precipitation. At bores between the infiltration basin and the sewer trench (mid region), groundwater quality can be attributed to the interaction of solute with the soil matrix, while for the three bores downstream of the sewer trench (DS72, Ref DS93 and DS48), the overall groundwater quality can be attributed to evaporation and its effect on concentration. These observations are in agreement with the results from the Piper diagrams (Fig. 5). Based on the results, the sampling sites could be, with a few exceptions, grouped into three unique clusters (Fig. 6).

Cluster 1, Near Basin: Basin and immediately downstream bore (DS0) are precipitation-dominated as indicated by Piper and Gibbs plots as well as confirmed through ionic correlations that can be explained by the large volumes of water infiltrating relative to the rain that falls at these locations.

Cluster 2, Mid-section: DS7–DS30 are largely ion exchange-dominated bores (Piper and Gibbs plots) and display some seasonal variations in the water quality. Bore DS24 is expected to behave similarly to the other bores (DS0–DS30) located in the rock exchange dominated cluster area, as shown in Gibbs plot, but it shows unique behaviour from every other bore in the cluster, and similar to the basin and to potable water. This might potentially be due to preferential flow paths for this bore or the bore being disconnected from surrounding soil during construction of the basin in 2011. Bore 17E presented with similar hydrochemistry as DS48, DS72 and DS93, all of which are further downstream and receive a very small amount of baseflow from the basin. It is possible that this bore is not getting much flow from the basin.

Cluster 3, Near Stream: Bores DS48, DS72, DS93 show a distinct evapo-concentration signal and higher levels for Ca, Mg and other major ions compared to all other bores. This is likely due to enhanced evaporation or water lost to the sewer trench (urban karst) or a combination of both, as most of the infiltrated water gets consumed by the trees between the basin and Basin-Olinda Road, which is located just downstream of DS48 (Western et al, 2021) or the urban karst (Poozan et al., 2022). These three bores also show high nutrient (nitrate) levels. This could be due to several reasons, such as decaying organic matter, wastewater leakage from the sewers or septic tanks, solid waste disposal (landfills and waste tips), use of fertilisers in the catchment or some other local source (Keeney & Olson, 1986; Wakida & Lerner, 2005).

While the tree density downstream of sewer trench and the zone between the basin and the sewer trench was the same, a much weaker evaporation signal was reported between basin and sewer trench. One potential reason might be that trees immediately downstream of the basin utilise a large fraction of the infiltrated stormwater and a very small fraction is available as baseflow further downstream (Western et al, 2021). This observation indicates that the subsurface environment is complex in urban areas and that vegetation, soil geology and disturbance to the subsurface through infrastructure and its associated trenches, have an important part to play. Additionally, the sewer trench (urban karst) located between DS30 and DS 48 might also divert some of the infiltered water (Poozan et al., 2022). Indeed, it is likely that multiple factors will simultaneously influence groundwater chemistry and behaviour.

### Chemical composition: Stiff pattern diagram

The Stiff diagram results show five distinct shapes / water types. Samples indicate changes in water quality between seasons for Basin, DS 0, DS 30, DS 72 and Dobsons Creek, (Fig. 3). DS 0, DS 17, DS 17W and DS 24 (near basin and mid cluster) showed a similar pattern, therefore only DS0 is displayed in Fig. 3. Also, DS 24 DS 48, DS 72 and DS 93 (near stream cluster) showed a similar pattern, therefore only DS72 is displayed in Fig. 3. The different colours in Fig. 3 represent the four distinct seasons of the year.

In the basin, calcium levels reduced from Aug–Sept (winter–spring) and then again from Jan–April (summer–autumn) due to reducing rainfall over each of these periods. DS0 showed uniform water quality throughout the sampling period. DS17E displayed Na/K–Cl type characteristics with fluctuations in chloride levels with season. DS30 showed Na–Cl type in winter but showed higher levels of calcium and bicarbonate with lower levels of chloride in January (summer).

DS48 was Na–Cl type in winter and moved to reduced chloride in November (spring). Levels of chloride increased in Dec (summer) and bicarbonate reduced to zero. DS72 was also Na–Cl type in winter, but in Nov (spring) and May (autumn) showed reduced levels of chloride and increased bicarbonate following the rainfall. DS93 displayed no change in chemistry until April/May (autumn), and then chloride levels reduced, and bicarbonate increased.

For ST West, the bore water quality during the first rainfall event in July (winter) was chloride dominant but changed to chloride deficient and bicarbonate rich in the summer. DS7 displayed a big change in October (spring) with magnesium and chloride levels increasing and bicarbonate levels decreasing.

Results from stiff diagrams show that most the bores displayed changes in water quality with season particularly in spring and summer. Bores DS 0, DS 17, DS 17W, DS 24, Dobsons Creek and ST East were the exception and displayed stable water quality. This might be potentially due to a preferential flow path to DS17, DS 17W and DS 24 that disconnected these bores from the rest of the bores as a result of infill after the basin was installed. The uniform water quality in DS 0 might be due to filter media in the basin filtering out most of the contaminants from infiltrated stormwater. DS 24 followed water quality trends similar to near stream bores, potentially due to preferential flow paths as explained above. The near basin cluster and mid-section (cluster 1 and 2) displayed varying levels of chloride, indicating varying levels of evapotranspiration, with reduced chloride/high bicarbonate in summer indicating new water/rainwater due to infiltration. The near stream cluster (cluster 3) on the other hand displayed reduced chloride levels in spring and increasing chloride/reducing bicarbonate in summer indicating strong evapo-concentration and little contribution from new water.

### Ionic correlations

Figure 5 a and b shows Na/Cl ratios greater than 1 and negative CA-1 values for bores DS0, DS7, DS17, DS 17W, DS30 and Dobsons creek. This indicates that the Ca and Mg ions in the water are exchanged with Na and K ions of the clays through indirect base exchange reaction. On the other hand, bores DS 48, DS72 and DS 93 show positive values for CA-1 values and Na/Cl ratios of less than 1, indicating a direct base exchange reaction (the exchange of Na and K ions from water with Mg and Ca ions of the rocks).

HCO_3_ + CO_3_/ Cl ratios greater than 1 (Fig. 5c) indicate shallow groundwater (freshwater input) for the basin, potable water and DS24 and ion exchange and/or evapo-concentrated water at DS7, DS 17, DS 17E, DS 30, DS 48, DS 72, DS93 and Dobsons Creek, in agreement with the results from piper and Gibbs plot.

The concentration of heavy metals were found to be low at all sampled locations and within permissible limits, likely because the study catchment is in peri-urban region, without significant industrial or other pollutant sources and that the infiltration basin removed all the heavy metals from infiltered stormwater. In more urbanised catchments where stormwater runoff can bring high levels of heavy metals, microplastics and other contaminants, the enhanced stormwater infiltration can introduce these contaminants, as well as mobilise legacy contaminants. The baseflow contribution is relatively small in the studied system (Western et al, 2021), but if the baseflow contribution were larger, this might have detrimental impact on the stream water quality, particularly during low flow periods. In high flow periods, where much of the flow is directly from stormwater, water quality will also be degraded (Makepeace et al., 1995).

Results showed that although the infiltrated stormwater did not present a contamination issue in the subsurface in the studied system, it has the potential to mobilise the existing contaminants from other sources present in the catchment, including potential legacy contaminant sites or potential continuous sources of contamination like leaky sewers or other localised sources. The infiltrated water can also interact with the soil matrix and leach out ions, which may pose a water quality threat to the intended downstream receiving water. The results show elevated levels of nitrate at some bores in a site that has never had any residential/industrial development on it. This indicates that the source of contamination might be quite distant from where the contaminants appear. This demonstrates that historical land-uses and pollutant sources alone cannot be relied upon to predict the presence and distribution of subsurface contamination and ongoing monitoring might be necessary.

At this site, dense vegetation downstream of stormwater infiltration basin can utilise most of the infiltrated water and thus result in only minimal contributions to baseflow, occurring only during the wetter periods where evapotranspiration is minimal. More generally, introduced contaminants can also be released into surrounding soils if the infiltration filter media is not properly maintained or replace after chemical exhaustion, through regeneration or replacement (Biswal et al, 2022). In this case, the infiltration basin substrate itself can become a source of contamination and can be, along with the legacy contaminants, flushed into receiving waters. It is important to understand the processes that control the fate and movement of infiltrated stormwater as well as solutes (both introduced and legacy) for optimal management of infiltration basins and the resultant water quality in surrounding groundwater and receiving waters.

Overall, the results suggest that the magnitude and quality of baseflows in urban regions depends significantly on the downstream vegetation, urban karst and the presence of legacy contaminants in the soils, as well as contaminants introduced along with stormwater. This needs to be considered in the design of stormwater infiltration basins, and in their siting relative to other land uses and potential legacy pollutant sources. Ideally, infiltration systems will only be constructed after a detail audit of potential pollutant sources, considering current and past land-uses, and sampling of groundwater (either from existing bores or purpose-constructed shallow groundwater bores).

## Conclusions

This paper used the differences in physico-chemical properties of water collected at different locations and times downstream of a stormwater infiltration basin and identified the origin and source of water and compared the processes driving that chemistry. A combination of graphical (Piper, Gibbs and Stiff plots) and correlational methods (several ionic ratios) was used to provide insights into water types and composition and processes and interactions with the soil. The spatial and temporal variability observed in the composition of major ions (natural tracers) provided useful insights into the origin of water, aquifer heterogeneity, connectivity and the physico-chemical processes controlling the groundwater chemistry downstream of the stormwater infiltration basin. The results indicated that not all bores exhibit the same behaviour due to differences in their location and immediate environment and were grouped into 3 clusters based on water quality. The results indicated that the magnitude and quality of baseflows in urban regions depend significantly on the downstream vegetation, urban karst and the presence of legacy contaminants in the soils, as well as contaminants introduced along with stormwater and therefore these factors must be considered in design phase.
